# Aqua Marina

**Published:** 1897-08

**Authors:** Thomas Skinner

**Affiliations:** London, England


### AQUA MARINA.

### A REMARKABLE CURE OF OPHTHALMIA.*

THOMAS SKINNER, M. D., LONDON, ENGLAND.

The subject of this case has never been seen by me, as she has never resided out of Australia (Sidney, N. S. W.), and I have never been nearer Australia than where I am now.

Mrs. S., an eminent artist, has been suffering from granular ophthalmia of her right eye, which has defied the best advice and services of a most excellent Hahnemannian physician, as also of an equally excellent allopathic oculist, for the last ten months. Of course, it is needless to say that the disease has interfered very considerably not only with her comfort, but also with her profession.

I am led to understand that the patient obtained no relief from the homœopathic treatment, and the allopathic treatment being limited to the usual caustic treatment and tonics, the amount of aggravation and amelioration can readily be guessed.

The patient writes to her sister in London: "After Friday's application of the irritant (5th March, 1897) the eyelid was so painful and swollen that I put a piece of raw veal on it when I got home from my visit to the oculist, about 2 P. M. All night also I kept applying raw veal, and I found immense relief to the pain and swelling caused by the caustic treatment. In twenty-four hours it was completely well, but the granular ophthalmia with its agglutination in the morning and its muco-purulent discharge continued the same as ever."

The application of raw veal was the patient's own prescription. She says in her letter to her sister: "I had read somewhere of Mrs. Langtry using it to *nourish the skin*, so I

---

* Read before The International Hahnemannian Association, June, 1897.

thought my weak eyelid might get some nourishment from it."

*Diagnosis of the remedy.* My only guide to the remedy was a fact, a condition of "*amelioration* to the affection, by *bathing the eye with sea-water, the only thing that ever relieved it.*"

As I have used sea-water (Aqua marina) for over twenty years in my practice for constipation aggravated or induced by a temporary residence at the sea-coast, also for any disturbance to the system resulting from a saline atmosphere, I had no difficulty in telling the sister that if she sent the following two powders to Mrs. S., and, if she took them as directed, they were qualified to do her sister no harm, if they did her no good.

*Prescription. Aqua marina* 20m (F. C.) two powders. One to be taken at bedtime on receipt, dry on the tongue. If no better in a week to dissolve the other in an ordinary hand-basin of cold or tepid water, and to hold her eyes open under the water as long as convenient once daily. After the second eye-bath she states: "I have now been over three weeks free from any inconvenience, and I have not had to wipe my eye constantly as formerly. I have been nearly ten months with a handkerchief never out of my hand."

She went back to her oculist a week after, and he said "It was *much* improved," and, when nearly a fortnight after, he said, "My word, it is *wonderfully better.*"

As the irritant or caustic treatment with tonics had been going on for months, it is safe to conclude that it had nothing to do with the certain and rapid cure. Lastly, Mrs. S. states that hitherto "after the irritant treatment, the discharge has always been plentiful for some days after." There has been little or none since the caustic treatment was stopped. The raw veal could only reduce the swelling, irritation, and pain induced by the cauterization of an already inflamed surface, and the conclusion I have come to is that the *Aqua marina* 20m (F. C.) was the therapeutic agent in the cure, and the keynote for *Aqua marina* in granular, scrofulous, or Egyptian ophthalmia is *amelioration from bathing or fomenting with sea-water.*

This medicament was made from the purest water of the Mediterranean Sea between Spain and Italy, after the complete subsidence of a storm, when it was "as clear as water, of the crystal spring."

